# Seasonal Photochemical Transformations of Nitrogen Species in a Forest Stream and Lake

**DOI:** 10.1371/journal.pone.0116364

**Published:** 2014-12-31

**Authors:** Petr Porcal, Jiří Kopáček, Iva Tomková

**Affiliations:** 1 Biology Centre of the Academy of Sciences of the Czech Republic, Institute of Hydrobiology, České Budějovice, Czech Republic; 2 Faculty of Science, University of South Bohemia in České Budějovice, České Budějovice, Czech Republic; University of Siena, Italy

## Abstract

The photochemical release of inorganic nitrogen from dissolved organic matter is an important source of bio-available nitrogen (N) in N-limited aquatic ecosystems. We conducted photochemical experiments and used mathematical models based on pseudo-first-order reaction kinetics to quantify the photochemical transformations of individual N species and their seasonal effects on N cycling in a mountain forest stream and lake (Plešné Lake, Czech Republic). Results from laboratory experiments on photochemical changes in N speciation were compared to measured lake N budgets. Concentrations of organic nitrogen (N_org_; 40–58 µmol L^−1^) decreased from 3 to 26% during 48-hour laboratory irradiation (an equivalent of 4–5 days of natural solar insolation) due to photochemical mineralization to ammonium (NH_4_^+^) and other N forms (N_x_; possibly N oxides and N_2_). In addition to N_org_ mineralization, N_x_ also originated from photochemical nitrate (NO_3_^−^) reduction. Laboratory exposure of a first-order forest stream water samples showed a high amount of seasonality, with the maximum rates of N_org_ mineralization and NH_4_^+^ production in winter and spring, and the maximum NO_3_^−^ reduction occurring in summer. These photochemical changes could have an ecologically significant effect on NH_4_^+^ concentrations in streams (doubling their terrestrial fluxes from soils) and on concentrations of dissolved N_org_ in the lake. In contrast, photochemical reactions reduced NO_3_^−^ fluxes by a negligible (<1%) amount and had a negligible effect on the aquatic cycle of this N form.

## Introduction

The photochemical properties of dissolved organic matter (DOM) have been thoroughly studied during last two decades, mostly focusing on the fate of organic carbon. The interest in chemical changes in surface water composition accompanying the photodegradation of DOM has gradually also included other environmentally important elements, like iron (Fe), aluminum (Al), nitrogen (N), and phosphorus (P) [1], [2], [3]. Of these, N transformations are the most complex, and may include photochemical cleaving of dissolved organic nitrogen (N_org_; a common constituent of DOM), resulting in the production of inorganic N forms, the oxidation of ammonium (NH_4_^+^), as well as the reduction of oxidized N forms (NO_2_^−^, NO_3_^−^) e.g., [4].

N_org_ is usually composed of a wide range of compounds ranging from low molecular weight amino acids and amino sugars to high molecular weight polyphenol-bound N [5]. Photodegradation of N_org_ produces certain forms of bio-available nitrogen, mainly NH_4_^+^ (a process called ‘photoammonification’) and amino acids [6]. Kieber et al. [7] have demonstrated that nitrite (NO_2_^−^) may be photochemically released from the nitroalkenes in humic substances through oxidation by singlet oxygen under natural sunlight. Photoproduction of ammonium has been observed from humic and fulvic acids in the Suwannee River [8], and in Baltic seawater [9]. Reactions of amino groups with hydroxyl radicals (HO·) are the dominant mechanisms of ammonium photoproduction e.g., [4] often enhanced by the presence of a catalyst (e.g., Fe or Ti oxides [10]).

Hydroxyl radicals, the most efficient oxidation agent, are produced in natural waters by several mechanisms including the direct photolysis of NO_3_^−^ and NO_2_^−^ e.g., [11], hydrogen peroxide, ozone e.g., [12], DOM e.g., [13], and FeOH^2+^ arising from the reaction of Fe^2+^ with H_2_O_2_ (the Fenton reaction) [14]. The percent contribution of HO· production from NO_2_^−^ and NO_3_^−^ ranged between 6–26% and 1–49%, respectively, in upstream and pristine rivers [11]. The HO· formed is easily scavenged by nitrite and organic matter, thus steady state concentrations depend on a combination of nitrate and DOM concentrations and the intensity of light e.g., [4].

The photochemical release of inorganic nitrogen from DOM (and especially photoammonification) is an important source of bio-available nitrogen in N-limited aquatic ecosystems, such as high-latitude or marine environments [3]. The effects of photochemical reactions on NH_4_^+^ concentrations in surface waters are not, however, unequivocal. While some studies have observed environmentally relevant rates of photoammonification e.g., [6], [9], [15], others have reported little or no NH_4_^+^ photoproduction e.g., [16], or even a photochemical loss of NH_4_^+^ [2], [17]. These contrasting results have been attributed to variations in DOM's intrinsic properties and prior light exposure history, environmental controls (e.g., pH and concentrations of Fe and dissolved oxygen), ambient concentrations of NH_4_^+^, and possibly methodological differences as well [18], [19]. Based on our previous results showing seasonal trends in the photochemical properties of DOM in headwater streams [20] and contradictory results of previous studies on photoammonification, we hypothesized that the N_org_ photoreactivity could exhibit a seasonal variability. The aim of this study was to conduct a series of irradiation experiments under constant conditions with water from a first-order forest stream to evaluate the seasonal photo-reactivity of DOM and N_org_. We used a simple pseudo-first-order kinetics model of N mass balance to quantify photo-induced changes in N speciation. In addition, we assessed the whole lake N mass budget, for all N species, including hydrological fluxes in a mountain forest watershed-lake system. Ultimately, these results provide insight into the significant photochemical transformations of N species in the context of N cycling in forest streams and lakes.

## Methods

### Description of study sites

**Figure 1:**
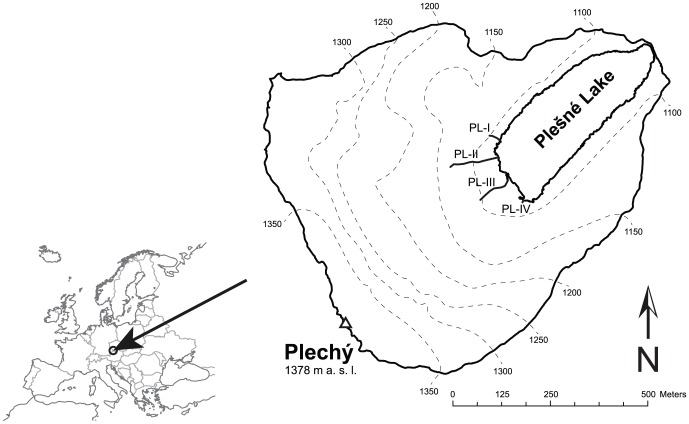
Watershed of Plešné Lake, its location in Europe, and locations of lake tributaries (PL-I to PL-IV) and throughfall precipitation plots (PL-H and PL-L). Bulk precipitation was sampled ∼0.4 km east of PL-L.

This study focused on the Plešné Lake and its major surface tributary (PL-I) (Fig. 1). Plešné Lake is situated in the Bohemian Forest (Czech–Austrian border, 48°47′N, 13°52′E) at an elevation of 1090 m. It is a dimictic lake of glacial origin with an area of 7.5 ha, maximum depth of 18 m, and theoretical water residence time of ∼300 days [21]. Watershed area is 66.6 ha (including the lake) and its maximum elevation is 1378 m. Annual average (± standard deviation) atmospheric water input to the watershed and runoff were 1332±178 and 1173±209 mm, respectively, during 2000–2013 [22]. The lake was already atmospherically acidified in the 1950s (pH<5.0), its acidification progressed until the middle of the 1980s, when pH decreased to ∼4.6, since then the lake has been recovering from acidification due to decline in acidic deposition [23]. The lake is fishless and zooplankton species are present in low densities [24].

Permission to access and collection of all samples was issued by the Administration of the National Park and Protective Landscape Area of Šumava (permission numbers: SZ NPS 06537/2009/4-NPS 0921/2009 and SZ NPS 09741/2011/4-NPS 00099/2012).

### Mass balance of nitrogen species and fluxes in the lake and its tributaries

Major fluxes of N forms (nitrate, NO_3_^−^; nitrite, NO_2_^−^; ammonium, NH_4_^+^; and dissolved organic nitrogen, N_org_) within the watershed-lake system (i.e., direct N deposition on the lake surface, terrestrial export via all tributaries, output, and net retention in the lake) were based on mass balances of water and its chemical constituents in the 2011 hydrological year (November 2010 to October 2011). NH_4_^+^ was determined by an ammonium specific colorimetric (rubazoic acid) method according to Kopáček and Procházková [25]. The original detection limit of the method (∼1 µmol L^−1^) was reduced to ∼0.3 µmol L^−1^ by using ultrapure water (freshly pumped through a mixed bed resin) for blanks. NO_3_^−^ and NO_2_^−^ (as well as other ions in this study) were determined by ion chromatography (Dionex IC25), with detection limits of ∼0.5 and 0.1 µmol L^−1^, respectively. N_org_ was calculated as the difference between Kjeldahl N and NH_4_^+^ concentrations, and Kjeldahl N was determined by Kjeldahl digestion, with 75 ml of samples evaporated to obtain a detection limit of ∼2 µmol L^−1^ [21]. Particulate N was the difference between total (unfiltered) and dissolved (filtered through 0.4 µm glass fiber filter; Macherey Nagel, MN-5, Germany) N_org_. The reliability of the analytical results was controlled by means of an ionic balance approach and a standard sample (a frozen subsample of stream water enriched with NH_4_Cl, which was melted and analyzed with each series of samples). Variability of N concentrations in the standard sample varied within ±3%, ±7%, and ±6% for average NO_3_^−^, NH_4_^+^ and N_org_ concentrations of 53, 7, and 37 µmol L^−1^, respectively, for 28 determinations in 2011.

The water balance was based on water input to the watershed and outflow from the lake. Water input to the watershed was determined from annual heights of precipitation measured at one treeless plot at elevation of 1087 m and at two throughfall plots situated in forest areas at low (1122 m) and high (1334 m) elevations. Outflow from the lake was continuously measured using a calibrated V-notch weir and gauge-recorder (recording in 15-min intervals). Actual discharges of tributaries were estimated using a stop-watch and bucket method. Annual water discharge from the watershed to lake by all tributaries was calculated from lake outlet and the budget for chloride (Cl^−^), assuming that Cl^−^ behaved conservatively with no net retention or production within the lake [26]. For more details see Kopáček et al. [21]. Chemical composition of atmospheric deposition used in this study was based on bulk precipitation measured in treeless area. Atmospheric deposition was sampled in 2–4-week intervals, tributaries in 3-week intervals, and the outlet in 1–3-week intervals. The fluxes of water constituents were obtained by linking discharge (inlet and outlet) and volume (precipitation) data with the corresponding concentration data, and net in-lake production (and/or retention) of the individual N forms was calculated similarly as in previous studies on Plešné Lake [21], [22].

The total uncertainties in the N fluxes were calculated using an error propagation method [27], assuming 3, 7, and 6% uncertainties in determination of NO_3_^−^, NH_4_^+^ and N_org_ concentrations. Uncertainty used for recording gauge measurements was 12%, based on CNI [28]. Uncertainty in the measured precipitation amounts was estimated as a variability of precipitation amount collected by 9 samplers and was 10% during 2010–2011.

### Photochemical experiments

Samples for photochemical experiments were collected from the major stream inlet (PL-I, Fig. 1) in three-week intervals from October 2010 to November 2011, with an additional sampling in March 2012. The PL-I tributary is a first order stream with its source ∼15 m from the lake and water residence time in minutes. Samples were collected into 20 L polyethylene terephthalate carboys previously washed thoroughly with acid and rinsed with demineralized water; additionally each carboy was rinsed with stream water before collecting. Samples were filtered through a 40 µm sieve to remove course particles re-suspended from the stream bed during collection, kept in the dark, immediately transported to the lab where they were filtered through a 0.4 µm glass fiber filter (Macherey Nagel, MN-5, Germany), and stored at 4°C before (up to one week) irradiation experiments.

For experiments, samples were divided into four aliquots (triplicates plus an unexposed control) and irradiated for time periods of 0 (start), 6, 12, 18, 24, and 48 hours in quartz tubes (25 cm length, 5 cm in diameter, total volume of ∼0.5 L) in an UV irradiation chamber (Rayonet RPR-200, The Southern New England Ultraviolet Company, USA). The UV irradiation chamber was equipped with sixteen UV lamps (Rayonet RPR-3500, The Southern New England Ultraviolet Company, USA) emitting radiation in the UV-A range with a maximum at 350 nm. The temperature of irradiated samples did not exceed 30°C which was approximately 5°C above ambient laboratory temperature. Unexposed controls were wrapped in aluminum foil and kept at the same temperature for the same amount of time as irradiated samples. All glassware and vials were previously washed thoroughly with acid and rinsed with demineralized water and additionally rinsed with sample.

**Figure 2:**
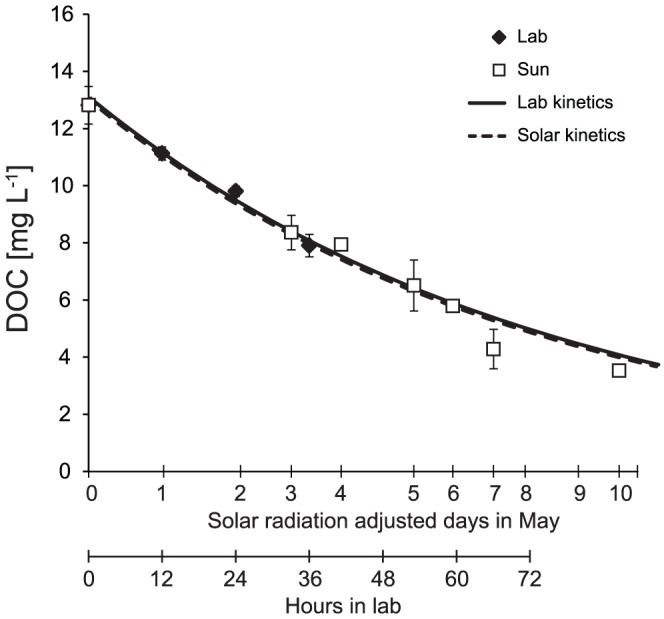
The decrease in DOC concentrations in the same sample exposed to laboratory UV irradiation (Lab) and natural solar radiation (Sun). The X-axis representing days of exposure to solar radiation (May 2012) was adjusted to the daily measured solar insolation at the Římov station.

A comparison between exposure to UV irradiation and natural solar irradiation was done by exposing the same water sample in quartz bottles to UV radiation in a UV irradiation chamber and to natural solar radiation in an open area (meadow) outside the Institute of Hydrobiology (48°58′N, 14°27′E). The intensity of solar insolation was measured irregularly during the exposure by a Digital Lux Meter AR823 (Smart Sensor, China) and compared with a continual pyranometer (CMP11, Kipp & Zonen, Netherlands) measurement from the Řiacute;mov meteorological station (14 km away; 48°51′N, 14°29′E). Irregular measurements correlated well (R^2^ = 0.81) with the continual data, which were thus used to estimate daily insolation energy doses. Fig. 2 shows the comparison between the decrease in DOC concentration during experiments with laboratory UV exposure and under natural solar radiation based on insolation energy doses (48 hours of exposure to the artificial UV radiation in the laboratory corresponded to ∼4–5 days of natural solar radiation in May 2012).

### Chemical and optical analysis

After irradiation, all aliquots were filtered through 0.4-µm glass-fiber filter to remove possibly formed particles [29] and analyzed for concentrations of N forms (as described above) and DOC and Fe using the following methods: DOC was analyzed by catalytic combustion at 680°C (Shimadzu 5000A, Japan) with a detection limit of ∼8 µmol L^−1^. Concentrations of Fe were determined by atomic absorption spectrophotometry (Varian AA240Z with a GTA 120 graphite tube atomizer) with a detection limit of ∼0.05 µmol L^−1^. Absorbance spectrums were measured in a 4 cm quartz cuvette from 200 to 800 nm (Specord 210, Analytik Jena, Germany) and the content of chromophoric organic matter (CDOM) was determined as the integrated absorbance between 250 and 450 nm with 1 nm step according to Helms et al. [30].

### Iron addition experiments

In addition to the regular experiments, the effects of increased Fe and DOM concentration on photochemical changes of NH_4_^+^ and NO_3_^−^ were tested with samples from April 2011 and October 2011. A solution of FeCl_3_ (6 mmol L^−1^) was used to increase the Fe concentration in the original sample from 2.5 to 30 µmol L^−1^. The dissolved organic carbon (DOC) and chromophoric dissolved organic matter (CDOM) concentrations were increased up to three times above their initial concentrations (i.e., DOC from 0.7 to 2.1 mmol L^−1^) by adding a freshly dissolved solution of humic acids isolated from the O soil horizon close to our sampling site at PL-I (HA1 isolate; for details see [1]. Amended samples were irradiated for 24 and 48 hours under the same conditions as the original samples, and the changes in NH_4_^+^ and NO_3_^−^ concentrations were determined.

### Quantification and modeling of nitrogen transformations during irradiation

**Figure 3:**
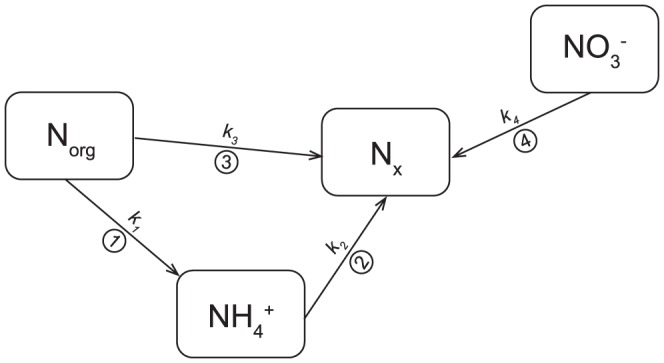
A scheme of possible transformations between nitrogen species. N_x_ represents the sum of undetermined nitrogen species (nitrogen oxides, N_2_, and NO_2_^−^) possibly formed during the photodegradation of major N forms. Abbreviations *k_1_* to *k_4_* represent pseudo-first-order photodegradation kinetics rate constants of reactions 1 to 4, respectively.

Seasonal variation in photochemical properties of DOM was characterized by changes in pseudo-first-order kinetics rate constants obtained from the nitrogen mass balance as follows: In each experiment, the initial concentration of total nitrogen, *c*(*TN*)*_0_*, was calculated as the sum of molar concentrations of individual nitrogen species according to equation (1):
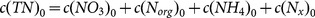
(1)where *c(NO_3_)_0_*, *c(N_org_)_0_*, and *c(NH_4_)_0_* are the initial concentrations of the measured nitrogen species, and *c(N_x_)_0_* is the initial concentration of all other unmeasured N forms (nitrogen oxides) and those with concentrations below the detection limit (NO_2_^−^). The *c(N_x_)_0_* values were considered negligible (*c(N_x_)_0_* = 0.0 µmol L^−1^) in natural samples prior to photochemical experiments. The N_x_ produced during the experiment at any time *t* [*c(N_x_)_t_*] was calculated as the difference between *c(TN)_0_* and concentrations of the measured N species (*c(NO_3_)_t_*, *c(N_org_)_t_*, and *c(NH_4_)_t_*) at time *t* (equation 2):
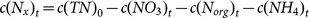
(2)The *c(N_x_)_t_* concentration includes all unmeasured nitrogen products potentially resulting from phototransformations of all N species, similarly to *c(N_x_)_0_* plus N_2_. Most of the individual members of equation (2) are affected by more than one process in parallel. The *c(NH_4_)_t_* concentration increases due to photoammonification (reaction 1 in Fig. 3). To explain the previously observed decrease in NH_4_^+^ concentrations during photochemical experiments [2], [17], we hypothesize for the purpose of this study that NH_4_^+^ could be partly oxidized to N_x_ (reaction 2 in Fig. 3). The *c(N_org_)_t_* concentration is reduced by the parallel production of NH_4_^+^ and NO_x_ (reactions 1 and 3 in Fig. 3). The *c(N_x_)_t_* concentration increases due to three fluxes: (i) oxidation of NH_4_^+^, which was either originally present in the sample or liberated from N_org_ by photoammonification, (ii) reduction of the original NO_3_^−^ (reaction 4 in Fig. 3), and (iii) directly from N_org_ oxidation.

A mathematical model using four differential equations (3–6) was used to describe changes in N speciation and compare measured and modeled results:
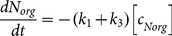
(3)

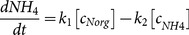
(4)

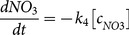
(5)

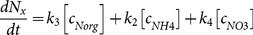
(6)where k_1_ to k_4_ represent pseudo-first-order photodegradation kinetics rate constants of reactions 1 to 4, respectively (Fig. 3). The rates of individual chemical reactions were not known, but were estimated using the pseudo-first-order reaction kinetics approach according to Stone and Morgan [31]. This method could be used because photochemical changes utilized only a small portion of individual species, which remained in excess over the reaction products. The first order kinetic curves were fitted through measured N species concentrations in time (0, 6, 12, 18, 24, and 48 hours of irradiation). The curves of modeled concentrations of N species at any time were determined by solving the differential equations (3–6) as follows:

The modeled NH_4_^+^ concentration after irradiation time *t* (*c(NH_4_)_t_*) was calculated as:
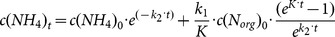
(7)where *t* is the time of irradiation in hours and *K* = *k_2_−k_1_−k_3_*, where *k_1_*, *k_2_*, and *k_3_* are pseudo-first-order kinetic rate constants (h^−1^) for reactions 1, 2, and 3, respectively, in Fig. 3.

N_x_ produced from NH_4_^+^ (*c(N_x_)_NH4_*) was calculated as the decrease in N_org_ concentrations via the NH_4_^+^ pathway (consecutive reactions 1 and 2 in Fig. 3) minus *c(NH_4_)_t_* according to the equation (8):
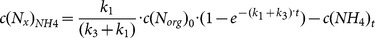
(8)where *c(NH_4_)_t_* was calculated from the equation (7).

The N_x_ formed directly from N_org_ [*c(N_x_)_Norg_*] via reaction 3 in Fig. 3 was calculated as:
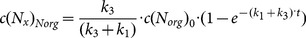
(9)The N_x_ formed directly from NO_3_^−^ [*c(N_x_)_NO3_*] was described by a pseudo-first-order kinetics equation (10):
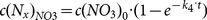
(10)where *k_4_* is the pseudo-first-order kinetic rate constant (h^−1^) for reaction 4 in Fig. 3 and *c(NO_3_)_0_* is the initial NO_3_^−^ concentration.

The kinetic rate constants, *k_1_*, *k_2_*, *k_3_*, and *k_4_*, were determined by the non-linear least square regression method by fitting measured data with the system of equations (7–10) [29]. The coefficient of determination (R^2^) between measured and modeled data ranged between 0.996 and 0.999. A 95% confidence interval (CI) for modeled data was calculated as:
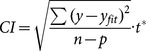
(11)where n represents degrees of freedom, p is a number of parameters determined during one regression and t* is the critical value for the t distribution with n – 1 degrees of freedom. Uncertainty propagation for modeled parameters was determined by fitting modeled data ± CI. All calculations were done in Microsoft Excel by the Solver Add-In. Basic statistics were calculated with Statistica 8.0 software (www.statsoft.com).

### Comparison of experimental changes in nitrogen speciation to their fluxes in natural surface waters

Laboratory results on photochemical changes in N speciation were compared to the measured N fluxes in Plešné Lake and its tributaries as follows: First, we estimated possible “natural” photochemical changes in N concentrations occurring during a one-day exposure to solar insolation, using equations (7–10). For each sampling period, the experimental irradiation time equivalent to the one-day solar insolation was estimated from a 30-year daily average of incident solar radiation for the study site (N.A.S.A. web page) and the measured relationship (Fig. 2) between laboratory UV irradiation and natural solar radiation (see above). The 30-year daily average was used because of the lack of continuous measurement of solar radiation near Plešné Lake. Second, the modeled concentration changes in N forms during the 3-week sampling periods were linked with the corresponding average discharges of tributaries expressed on a lake-area basis (L m^−2^ d^−1^) [22], and then summed for the whole ice-free period (4 April 2011 to 21 November 2011).

## Results

### Chemical changes during irradiation

**Figure 4:**
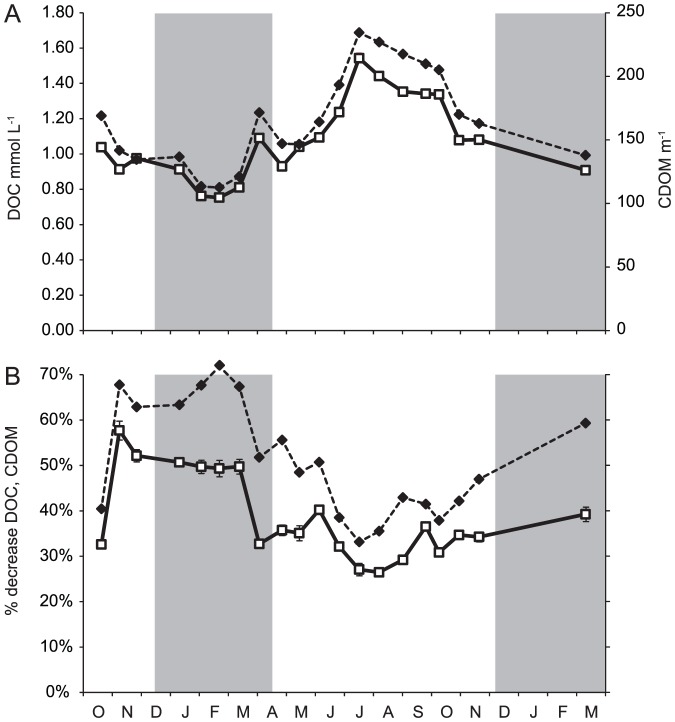
Seasonal trends in (A) initial DOC and CDOM concentrations (dashed line - calculated as the sum of absorbance from 250 to 450 nm according to [30]) in stream water, and (B) percent decrease in DOC and CDOM (dashed line) after 48 hours of irradiation. Grey areas represent the snow cover periods. Error bars represent 1 standard error of triplicate samples.

The average initial concentration of DOC in the stream water used for irradiation experiments was 1.08 mmol L^−1^ and ranged from 0.75 to 1.54 mmol L^−1^, with the highest and lowest values in summer and winter, respectively (Fig. 4A; for other chemical parameters see Table 1). Photochemical degradation of DOM after the 48-hour irradiation resulted in a 26–58% (39% on average) decrease in DOC concentrations, with the maximum and minimum changes in November 2010 and August 2011, respectively (Fig. 4B). The seasonal trends in DOC and CDOM concentrations and their relative changes during irradiation exhibited inverse patterns, i.e., higher photochemical degradation occurred in samples with lower CDOM (Fig. 4). This inverse relationship could in part be accounted for by self-shading during photochemical experiments. In this study, however, the light pathway was short (irradiation tubes with 5 cm diameters) and the shading effect was probably small (15% according to the calculation: (1-e^−a350×path length^)/(a_350_×path length), where a_350_ is the absorption coefficient and path length is the average distance light traveled through the solution = 2.5 cm [32]).

**Table 1:** Initial chemical composition of stream samples (minimum, mean ±1 standard error, maximum).

	minimum	mean ± error	maximum
pH	4.17	4.20±0.03	4.27
DOC	0.75	1.08±0.22	1.54
N_org_	40	48±17	58
NH_4_^+^	0.3	0.8±0.3	1.4
NO_3_^−^	58	138±59	205
NO_2_^−^	B.D.L.	B.D.L.	B.D.L.
Na^+^	30	49±8	61
K^+^	26	34±5	42
Ca^2+^	19	24±4	32
Mg^2+^	7	10±2	14
SO_4_^2−^	19	28±4	33
Fe	2.0	2.6±0.5	3.5

Units are µmol L^−1^, except for DOC (mmol L^−1^) and pH; B.D.L. - below detection limit.

**Figure 5:**
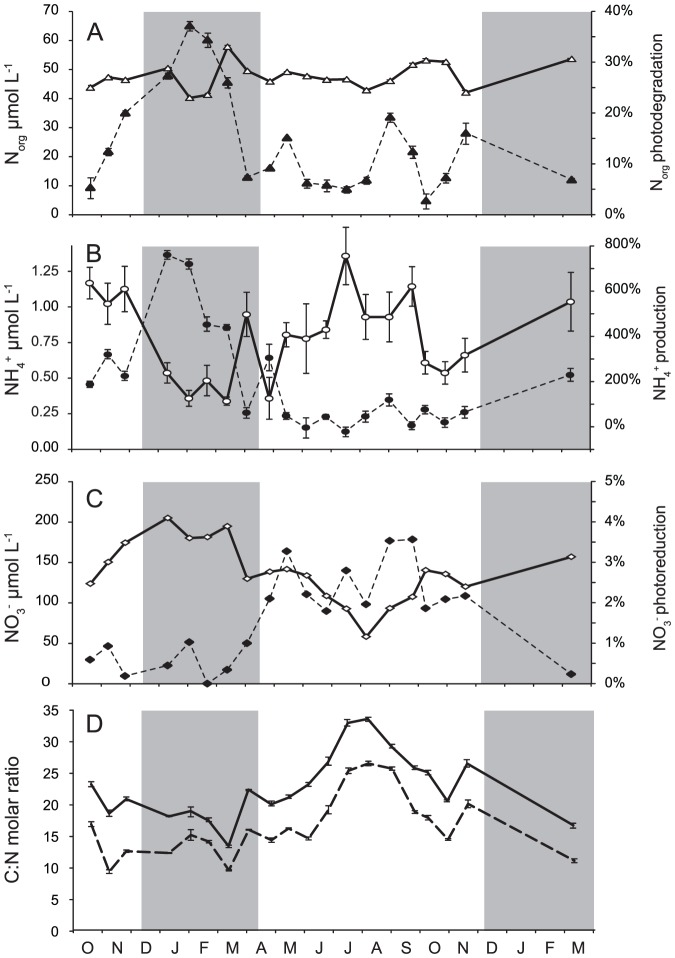
Seasonal changes in initial N_org_ (A), NH_4_^+^ (B), NO_3_^−^ (C) concentrations (white symbols) and percent change (black symbols) in N_org_ (A), NH_4_^+^ (B), NO_3_^−^ (C) concentrations after 48 hours of irradiation in stream water. C∶N molar ratios (D – no symbols), solid line represents C∶N ratio at the beginning and dashed line at the end of irradiation. Grey areas represent the snow cover periods. Error bars represent 1 standard error of triplicate samples.

The decrease in DOC concentrations was accompanied by a simultaneous decrease in the concentrations of N_org_. The annual average N_org_ concentration was 48 µmol L^−1^ and ranged from 40 to 58 µmol L^−1^ (Fig. 5A). The photochemical decrease rate observed in N_org_ was 0.031 to 0.314 µmol h^−1^, resulting in a 3 to 26% decrease after the 48-hour irradiation with an annual average loss of 11%. Molar C∶N (DOC∶N_org_) ratio ranged from 13.4 to 33.6 with annual average of 22.8. C∶N ratio exhibited a seasonal trend with minimum in winter and maximum in summer (Fig. 5D). The photochemical degradation of DOC was faster than photodegradation of N_org_ documented by decreasing C∶N ratio. After 48 hours of irradiation, the C∶N ratio decreased 17 to 61% (Fig. 5D).

The decline in N_org_ concentrations contributed to the production of NH_4_^+^ and N_x_. Because part of the N_x_ production originated from NH_4_^+^ photooxidation, the resulting net changes in NH_4_^+^ concentrations varied widely from 21% consumption to 760% production after irradiation, compared to their initial values of 0.3–1.4 µmol L^−1^. The rate of these changes ranged from −0.006 to 0.085 µmol h^−1^ with the decrease in NH_4_^+^ concentration observed in summer, while the maximum net NH_4_^+^ production occurred in winter (Fig. 5B).

Relatively small changes (0.2 to 3.5% decrease) occurred in NO_3_^−^ concentrations during the 48-hour irradiation. Even these small decreases, however, represented the dominant NO_x_ source throughout almost the whole study, because NO_3_^−^ was the dominant N form in the stream water, with concentrations ranging from 58–205 µmol L^−1^ (Fig. 5C). The highest decrease in NO_3_^−^ was observed in months without snow cover, while the lowest decrease occurred in winter, when the watershed was covered with snow (Fig. 5C).

The total photochemical production of N_x_ was not directly measured but was calculated from equation (2). After 48 hours of irradiation, the N_x_ production ranged from 2.6 to 24.9 µmol L^−1^ corresponding to 2–11% of the total nitrogen concentration, with annual average of 12.4 µmol L^−1^ (7% of the total nitrogen concentration). The N_x_ production during irradiation was the highest in winter and spring samples and the lowest in summer samples.

### First-order kinetics rate constants

**Figure 6:**
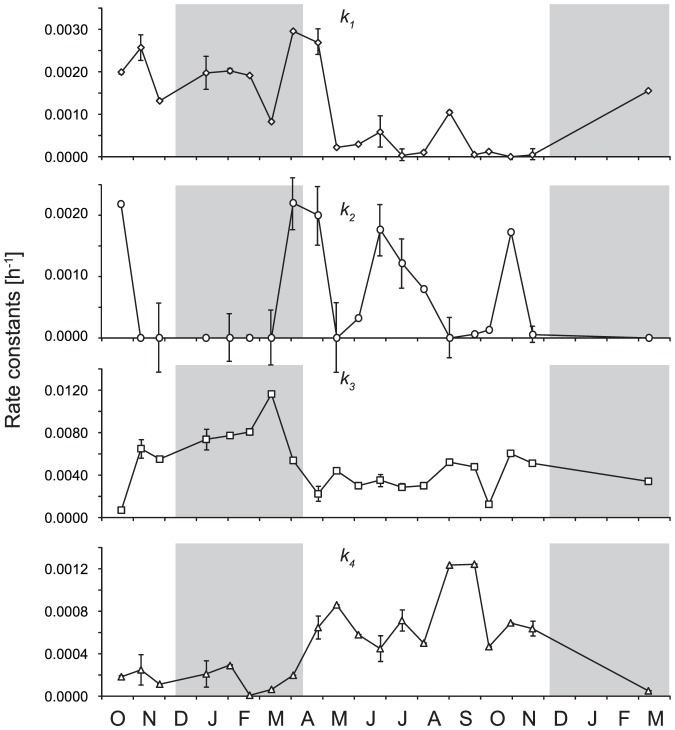
Modeled changes in pseudo-first-order kinetics rate constants of photoammonification (*k_1_*), subsequent oxidation of ammonia to N_x_ (*k_2_*), direct N_org_ transformation to N_x_ (*k_3_*), and NO_3_^−^ reduction to N_x_ (*k_4_*), respectively. Grey areas represent the snow cover periods. Error bars represent uncertainty propagation.

**Figure 7:**
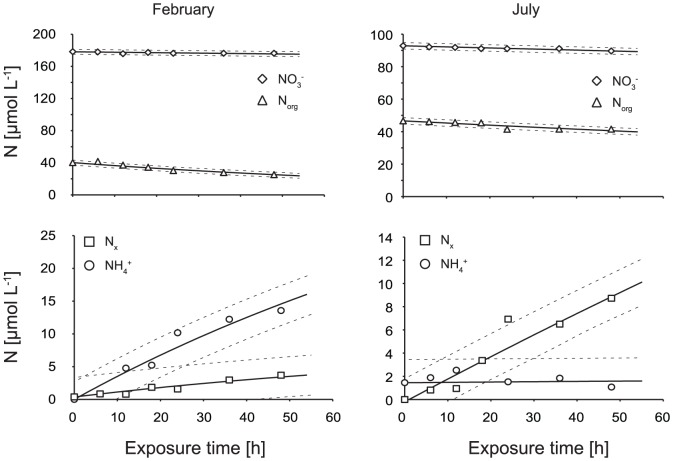
Measured and modeled changes in concentrations of NO_3_^−^, N_org_, NH_4_^+^, and N_x_ in irradiation experiments from February 2, 2011 and July 18, 2011, respectively. Points represent measured data, solid lines represent modeled concentrations, and dashed lines represent 95% confidence intervals. The y axis values differ between months.

The modeled first-order kinetics rate constants (Fig. 6) enabled a direct comparison between photochemical transformations of individual N species involved in N transformations. Fig. 7 shows measured and modeled concentrations of N species for a typical winter and summer irradiation experiments. The rate constant *k_1_*, representing the release of NH_4_^+^ from N_org_, was the highest immediately before and during the spring snow melt, then decreased and stayed low until the next winter. The rate constant *k_2_*, representing the consecutive consumption of formed NH_4_^+^, was low during winter and with increased values in spring and summer, when its values were higher than *k_1_*, resulting in NH_4_^+^ decrease. The rate constant *k_3_*, representing the direct oxidation of N_org_ to N_x_, consistently increased during the autumn and winter, and decreased immediately before and during snow melt and remained stable during the summer. On the other hand, the rate constant *k_4_*, representing the photochemical reduction of NO_3_^−^, exhibited the lowest values in winter and the highest in summer (Fig. 6).

There was a statistically significant correlation between the modeled first-order kinetics rate constants and chemical and meteorological parameters (Table 2). Rate constants *k_1_* and *k_3_*, describing photochemical degradation of N_org_, positively correlated with snow cover and NO_3_^−^ concentration, and negatively with DOC, CDOM, and total iron. The experimental doubling of iron concentration (as FeCl_3_) increased NH_4_^+^ photoproduction by ∼30% in the additional experiments (n = 4, p<0.05). Similarly, the experimentally increased DOC and CDOM concentrations resulted in higher photoammonification (n = 6, p<0.05). These additional experiments confirmed that a higher amount of hydroxyl and organic radical sources enhanced photoammonification.

**Table 2:** Spearman R correlations between pseudo-first-order photodegradation kinetics rate constants (*k_1_* to *k_4_*) and chemical and meteorological parameters (* p<0.05; ** p<0.01; *** p<0.001).

n = 20	DOC	CDOM	NO_3_^−^	Fe	Snow
*k_1_*	−0.60**	−0.51*	0.47*	−0.60**	0.50*
*k_2_*	−0.37	−0.37	0.21	−0.32	0.25
*k_3_*	−0.57**	−0.62**	0.59**	−0.57**	0.63**
*k_4_*	0.60**	0.57**	−0.57**	0.60**	−0.68***

### Modeled effects of one-day exposure to solar insolation

**Figure 8:**
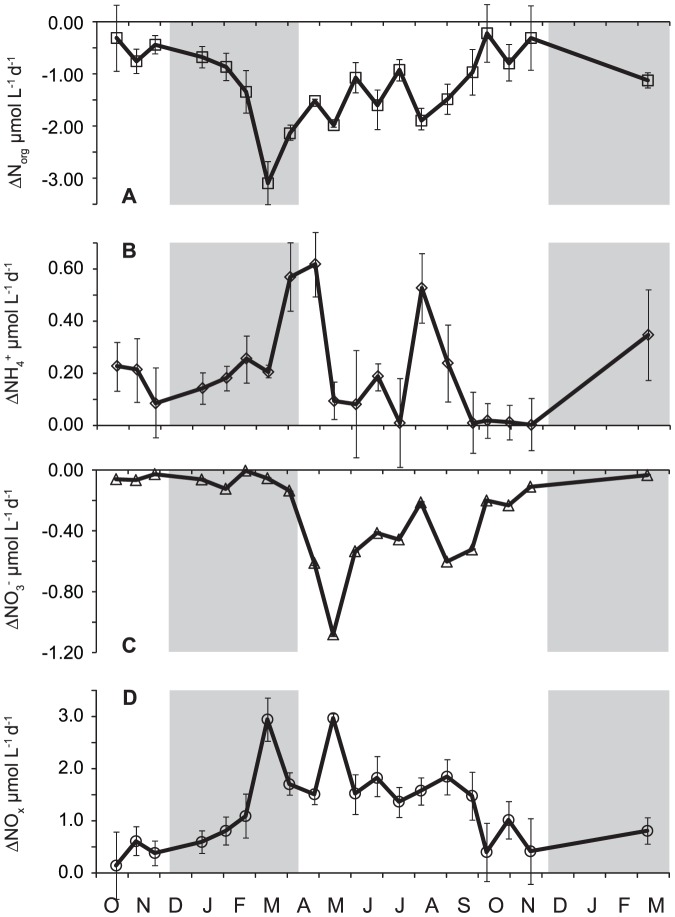
The calculated changes in N_org_, NH_4_, NO_3_, and N_x_ concentrations after one day of average solar insolation for given day. A - decrease in N_org_ concentration; B - increase in NH_4_ concentration; C - decrease in NO_3_ concentration; D - increase in other inorganic nitrogen. Error bars represent 1 standard error.

The computed one-day exposure to solar insolation from initial condition resulted in (i) a decrease in dissolved N_org_ by 0.2–2.1 µmol L^−1^ d^−1^ with maximum at the end of winter (Fig. 8A), (ii) an increase in NH_4_^+^ by 0.01–0.62 µmol L^−1^ d^−1^ with maximum at the end of winter (Fig. 8B), (iii) a decrease in NO_3_^−^ by 0.1–1.1 µmol L^−1^ d^−1^ with higher rates in spring and summer than in winter (Fig. 8C), and (iv) a 0.1–3.0 µmol L^−1^ d^−1^ production of N_x_ (Fig. 8D). The linking of these concentration changes with the corresponding average discharges of tributaries for the whole 242-day ice-free period showed that the cumulative changes in N speciation resulting from one-day solar insolation were a production of 97 mol NH_4_^+^ and a removal of 131 and 501 mol NO_3_^−^ and dissolved N_org_, respectively. On a lake-area basis, these changes represent an average daily production (± uncertainty) of 5.4±0.7 µmol m^−2^ d^−1^ NH_4_^+^ and a removal of 7.3±0.9 and 28±4 µmol m^−2^ d^−1^ NO_3_^−^ and dissolved N_org_, respectively.

### Major fluxes of N forms in Plešné Lake and its tributaries

On hydrologic year basis (November 2010 to October 2011), the daily average (± uncertainty) loading of Plešné Lake with individual N forms via tributaries was 10±1.4, 3013±373, and 573±77 µmol m^−2^ d^−1^ for NH_4_^+^, NO_3_^−^, and dissolved N_org_, respectively (Table 3). During the ice free period, the average NH_4_^+^ loading was 4±0.6 µmol m^−2^ d^−1^, i.e., 60% lower than the values based on the annual budget, NO_3_^−^ loading (3095±383 µmol m^−2^ d^−1^) was similar to the annual average, and the average loading of dissolved N_org_ (736±103 µmol m^−2^ d^−1^) was 28% higher than its annual average.

**Table 3:** Concentrations and fluxes of N species in precipitation (direct atmospheric bulk deposition on the lake surface), watershed export (via tributaries), and output from Plešné Lake in the 2011 hydrological year.

N-species1)	Precipitation (deposition on lake)		Watershed export (input by tributaries)		Output from lake		Change in storage2)	Net in-lake production3)
	µmol L^−1^	µmol m^−2^ d^−1^	µmol L^−1^	µmol m^−2^ d^−1^	µmol L^−1^	µmol m^−2 ^d^−1^	µmol m^−2 ^d^−1^	µmol m^−2 ^d^−1^
NH_4_^+^	19	53±7	0.6	10±1	3.9	75±10	−194±23	−183±26
NO_3_^−^	19	56±6	183	3013±373	100	1939±240	−302±9	−1432±443
N_org_	13	38±8	35	573±77	24	476±64	−89±5	−224±100
PN	7.2	21±5	1.2	19±5	26	505±146	149±6	614±147
TN	58	168±18	219	3615±444	154	2994±274	−436±25	−1225±523

Uncertainty (±) of the fluxes was calculated using an error propagation method [27].

1) Ammonium (NH_4_^+^), nitrate (NO_3_^−^), dissolved organic N (N_org_), particulate organic N (PN = total N_org_−N_org_), and total nitrogen (TN = NH_4_^+^+NO_3_^−^+N_org_+PN).

Concentrations of nitrite were <0.1 µmol L^−1^ and were neglected.

2) Change in storage represents the average change in amount of N species in the lake during study period and was based on differences between their concentrations at 0.5, 4, 9, 14, and 17 m depths on 31 October 2011 and 20 October 2010, and volumes of the respective water layers [21].

3) Net in-lake production was calculated on an annual basis as described by Kopáček et al. [21] (production = output+change in storage−input by precipitation and tributaries), and then recalculated to daily fluxes given on a lake-area basis.

On an annual basis, Plešné Lake was a net sink of NH_4_^+^, NO_3_^−^ and dissolved N_org_, and a net source of particulate N in the 2011 hydrological year (Table 3). Concentrations of NH_4_^+^ were 0.6 µmol L^−1^ on average in tributaries, and direct atmospheric deposition on the lake surface (on average 53±7) was the major ammonium source for the lake. In contrast, lake inflows were the dominant source of NO_3_^−^ and dissolved N_org_ for the lake, while their atmospheric inputs were an order of magnitude lower. In-lake processes removed on average 1430 and 220 µmol m^−2^ d^−1^ of the total NO_3_^−^ and dissolved N_org_ inputs to the lake, which represents 47% and 37%, respectively, of, their total inputs to the lake (Table 3).

## Discussion

### Changes in DOC and N concentrations during irradiation

The changes in DOC concentrations were accompanied by parallel, but more pronounced decreases in CDOM values (32–75%, Fig. 4B). Similar seasonal trends in DOC and CDOM photoreactivity, with maximum photodegradation during the spring snowmelt, were observed elsewhere (e.g., in humic first order streams in Canada [20]).

Our results on photoammonification (flux 1 in Fig. 3) showed an increasing trend in NH_4_^+^ concentrations (Fig. 5B) and maximum rates of pseudo-first-order kinetics rate constant *k_1_* (Fig. 6A) during irradiation of winter samples, while low values or even decreases in NH_4_^+^ concentrations occurred in summer samples. The net NH_4_^+^ photoproduction rates in this study (−0.006 to 0.085 µmol h^−1^) were within a wide range of similar data observed elsewhere, varying from photochemical loss of NH_4_^+^ in shallow ground waters (from −0.300 to −0.020 µmol h^−1^ [17]) to NH_4_^+^ production in a humic rich lake in Venezuela (0.060–0.130 µmol h^−1^ [33]) and in natural waters with high humic content (0.040–0.370 µmol h^−1^ [6]). The high variability in photoammonification was documented also by Stedmon et al. [9] and Aarnos et al. [15] who observed production; Wiegner and Seitzinger [16] who observed stagnation, and Vähätalo et al. [2] who observed decrease in NH_4_^+^ concentrations after exposure to solar or artificial radiation. Two reactions involved in net NH_4_^+^ production were used to explain this wide range of results. The first reaction, ammonification, represents the release of NH_4_^+^ from N_org_. The second reaction describes the oxidation of NH_4_^+^ to other N species, collectively expressed as N_x_ (equation 6 and flux 2 in Fig. 3). A net result of these processes probably reflects water composition and its seasonal changes.

The decrease in N_org_ concentrations was higher during irradiation than the sum of net changes in NH_4_^+^ concentration (associated with ammonification) and the N_x_ production (associated with NH_4_^+^ oxidation), i.e., with fluxes 1 and 2 in Fig. 3. This discrepancy can be explained by the direct N_org_ decomposition to N_x_ (flux 3 in Fig. 3), bypassing the NH_4_^+^ intermediate. This process has been observed in isolated humic substances, where NO_2_^−^ may be photochemically released from the nitroalkenes in humic substances through oxidation by singlet oxygen under natural sunlight [7]. A similar oxidation of N_org_ was also observed in fog water [34]. The proportion between these two N_org_ photodegradation pathways is given by the ratio of corresponding rate constants (i.e., *k_1_* to *k_3_* ratio, Fig. 3) with a median value of 0.22, which suggests that the direct N_org_ oxidation to N_x_ dominated the N_org_ removal in our study. Two N_org_ photodegradation pathways were observed also by Kieber et al. [7], but the NH_4_^+^ pathway dominated in their experiments with humic isolates from coastal, estuarine and fresh waters.

**Figure 9:**
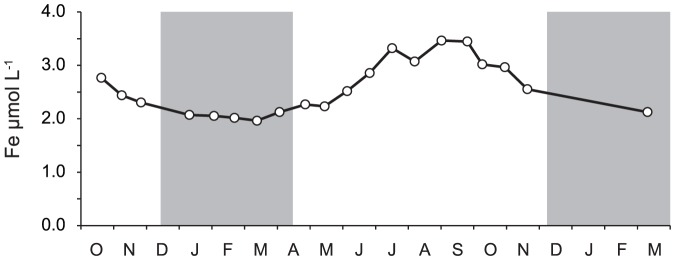
Concentration of iron in stream water used for photochemical experiments. Grey rectangles represent periods of snow cover. Error bars represent 1 standard error.

The most probable pathway of the NO_3_^−^ decrease is its reduction by the hydroxyl radical or Fe(II) to NO_2_^−^ and further to N oxides and N_2_ e.g., [10]. We observed an opposite trend between rate constants describing N_org_ decreases (*k_1_* and *k_3_*) and the photochemical reduction of NO_3_^−^ (*k_4_*). Nitrate photoreduction was highest in the summer months (Figs. 5 and 6) and positively correlated with DOC, CDOM and total iron concentrations (Table 2). The seasonal trend in iron concentration paralleled the trend in DOC and CDOM concentrations (Figs. 4 and 9), because most iron in the study stream is organically bound [22]. The additional experiments with increased Fe concentration confirmed increased NO_3_^−^ photoreduction (n = 12, p<0.05). On the other hand, the NO_3_^−^ concentration had the opposite trend to DOC and Fe concentrations, with the maximum NO_3_^−^ values in winter and minimum in summer. The higher nitrate photoreduction in summer despite a lower initial NO_3_^−^ concentration suggests that nitrate photoreduction by DOM [35] and/or nitrate reduction by Fe(II) produced by photo-Fenton reaction [10] played more important roles than direct nitrate photoreduction and HO· production.

### Photochemical transformation of N species

Our modeling of nitrogen transformations during irradiation encompassed four main fluxes (N_org_, NH_4_^+^, NO_3_^−^, and N_x_), characterizing changes in nitrogen speciation (Fig. 3). The use of modeled first-order kinetics rate constants is a useful approach in describing seasonality because the differences in initial concentrations are normalized to rates [31].

The net NH_4_^+^ photoproduction depends on the actual values of *k_1_* and *k_2_* and concentrations of N_org_ and NH_4_^+^. Values of *k_1_* were higher in winter than in summer (Fig. 6) and N_org_ concentrations were relatively stable throughout the year (Fig. 5A). The NH_4_^+^ production from N_org_ thus reached maximum rates in winter. In addition, the initial NH_4_^+^ concentrations were higher in summer than in winter (Fig. 5B), and *k_2_* values were usually higher than *k_1_* values during summer (Fig. 6), resulting in higher NH_4_^+^ consumption in summer than in winter. Both processes thus contributed to the higher net NH_4_^+^ photoproduction in winter than in summer (Fig. 5B).

Observed C∶N molar ratio exhibited strong seasonal trend with lower values in winter suggesting more nitrogen rich DOM than in summer, which is in concordance with observation of Jones et al. [36] who hypothesize that there are two distinct N_org_ pools in soils. The first pool comprises low molecular weight N_org_ species, mainly free amino acids and proteins, and is turned over very rapidly by the microbial community and thus does not accumulate in the soil. The second pool is formed by high molecular weight humic substances, which turns over slowly and represents the major N_org_ losses to freshwaters. The DOM transported to surface waters thus probably contains more photolabile low molecular weight N_org_ species during the winter and snowmelt period than in summer. Late winter is characterized by base flow, while during the spring snow melt flow reaches the highest values of the season, followed by its decline during rest of spring and summer. It has been observed that soil microbial biomass reaches an annual peak toward the end of the cold season, followed by a decline at the winter–spring transition, caused in part by a shortage of C substrates [37]. Co-incident with this decline in microbial biomass is the release of available soil N species, which peaks in late winter and decreases in early spring in seasonally snow covered ecosystems in the Rocky Mountains, U.S.A. [38]. Much of this pulse of available N is presumed to come from the lysis of cells of the late winter microbial flora that are nutrient-limited and intolerant of freeze-thaw events [39].

### Effect of increased DOC and Fe concentrations

Our results showed that experimentally increased concentrations of iron (up to twelve times) and DOC (up to three times) increased photoammonification and net NH_4_^+^ photoproduction. The seasonal cycle of DOC, CDOM (Fig. 4) and iron (Fig. 9) concentrations, with minimum values in winter and maximum in summer, resulted in negative correlations between these variables and the observed kinetics rates (Table 2). This disproportion between the observed seasonal trends and the positive effect of Fe and DOC additions on photoammonification in laboratory experiments suggests seasonal changes in the quality and photolability of DOM and N_org_ transported from the soil.

### Effects of photochemical transformations of N species on their fluxes in forest streams and lakes

The ecological importance of photochemical changes on N fluxes in surface waters differed for individual N species from negligible changes (NO_3_^−^) to a significant increase (NH_4_^+^) or removal (N_org_), and the relative importance of these changes for aquatic ecosystems further differed between the lake and its inlets.

Biological assimilation and denitrification were the dominant in-lake sinks of NO_3_^−^, similarly to previous years [21], [22]. Photochemical cleaving and, consequently, elevated availability of terrestrial N_org_ for the microbial community were probably the major reasons for the net removal of dissolved N_org_ (Table 3) as observed elsewhere [2], [40], [41]. In contrast, the in-lake primary and microbial production of new biomass [21] and, possibly, also photochemical mechanism of particle production [29] resulted in a net production of particulate N (Table 3).

The effect of the one-day solar insolation on the inlet water was only equivalent to <1% of the average NO_3_^−^ flux in tributaries (Table 3), and its net removal in the lake by assimilation and denitrification (Table 3) was 2–3 orders of magnitude higher than the photochemical removal (on average 1432 vs. 7 µmol m^−2^ d^−1^). The ecological effect of photochemical reactions on NO_3_^−^ fluxes is thus negligible for both forest streams and lakes.

The net photochemical production of NH_4_^+^ in the inlet water resulting from the one-day solar insolation represented a significant ammonium source, exceeding the average terrestrial export of NH_4_^+^ to the Plešné tributaries (5.4±0.7 vs. 4.0±0.6 µmol m^−2^ d^−1^). This photochemical NH_4_^+^ production could represent an ecologically significant ammonium source for microbial production in forest streams in N-limited systems, and especially in winter and spring, i.e. during periods with low NH_4_^+^ leaching from soils (Fig. 5B) and higher susceptibility of the terrestrial organic matter to photodegradation and photoammonification (Figs. 4B and 5B). In contrast to inlet streams, the relative importance of photoammonification was negligible for Plešné Lake, with the total NH_4_^+^ inputs dominated by direct atmospheric deposition (53 µmol m^−2^ d^−1^; Table 3). But, even though photoammonification represented a one order of magnitude lower NH_4_^+^ source than atmospheric deposition on a whole lake-area basis, this process may importantly contribute to NH_4_^+^ availability in lakes close to the effluents of tributaries, and especially during dry periods with low atmospheric deposition.

The effect of the one-day solar insolation on the inlet water was equivalent to ∼4% decrease in the average flux of dissolved N_org_ exported to the lake by tributaries (736 µmol m^−2^ d^−1^) during the ice-free period. The ecological effect of short-term solar radiation on concentrations of dissolved N_org_ in streams is thus probably negligible. But, the observed average in-lake removal of dissolved N_org_ was one order of magnitude higher than the simulated one-day effect (224±100 vs. 28±4 µmol m^−2^ d^−1^). This disproportion suggests that the effect of biogeochemical transformations of N_org_ is probably greater in lakes than in tributaries. The reasons behind this difference are (i) longer water residence time and exposure to solar radiation in the lake and (ii) a partial photochemical cleaving of terrestrial recalcitrant N_org_ to lower-molecular-weight particles available for the aquatic microbial community [2], [41]. The higher in-lake removal of dissolved N_org_ thus integrates a more pronounced direct photochemical mineralization with the effect of photochemically increased N_org_ availability for microbial uptake.

The seasonality in photochemical reactivity of N_org_ (Fig. 5A) probably reflected seasonality in the composition of N_org_ exported from soils. This result implies the need for future research, which should focus on the photochemical reactivity and composition of N_org_ isolated from different soils during vegetation and dormant periods.
